# Multi-Sensor Information Fusion for Optimizing Electric Bicycle Routes Using a Swarm Intelligence Algorithm

**DOI:** 10.3390/s17112501

**Published:** 2017-10-31

**Authors:** Daniel H. De La Iglesia, Gabriel Villarubia, Juan F. De Paz, Javier Bajo

**Affiliations:** 1 Computer and Automation Department, University of Salamanca, Plaza de la Merced s/n, 37002 Salamanca, Spain; gvg@usal.es (G.V.); fcofds@usal.es (J.F.D.P.); 2 Artificial Intelligence Department, Polytechnic University of Madrid, Campus Montegancedo s/n, Boadilla del Monte, 28660 Madrid, Spain; jbajo@fi.upm.es

**Keywords:** intelligent transport systems, information fusion, vehicular sensor network, energy efficiency

## Abstract

The use of electric bikes (e-bikes) has grown in popularity, especially in large cities where overcrowding and traffic congestion are common. This paper proposes an intelligent engine management system for e-bikes which uses the information collected from sensors to optimize battery energy and time. The intelligent engine management system consists of a built-in network of sensors in the e-bike, which is used for multi-sensor data fusion; the collected data is analysed and fused and on the basis of this information the system can provide the user with optimal and personalized assistance. The user is given recommendations related to battery consumption, sensors, and other parameters associated with the route travelled, such as duration, speed, or variation in altitude. To provide a user with these recommendations, artificial neural networks are used to estimate speed and consumption for each of the segments of a route. These estimates are incorporated into evolutionary algorithms in order to make the optimizations. A comparative analysis of the results obtained has been conducted for when routes were travelled with and without the optimization system. From the experiments, it is evident that the use of an engine management system results in significant energy and time savings. Moreover, user satisfaction increases as the level of assistance adapts to user behavior and the characteristics of the route.

## 1. Introduction

The success achieved in the field of hybrid electric vehicles (HEV’s) [1] and plug-in hybrid electric vehicles (PHEV’s) [2] has led to the production of 100% electric vehicles (EV-type). However, if we want to end our dependence on fossil fuels effectively, more research still needs to be done. When working on the development of electric vehicles, researchers focus on two clearly defined fields of study. First of all, it is necessary to concentrate on the physical development of the batteries which store electrical energy; one of the solutions we can consider is the introduction of new components, such as graphene [3] or hydrogen fuel cells [4,5]. A second field of study involves the development of management algorithms and the optimization of vehicle energy consumption at route segments. In this field, the fusion of information from open data sources should be considered, since this can help generate optimal configurations for EV route planning. Diverse sources of information can be used: road status, GPS (global positioning system), altitude or data obtained by the vehicles of other users (e.g., the average time taken to travel a route). Many studies have already focused on optimizing fuel and battery consumption in hybrid electric vehicles or plug-in hybrid electric vehicles [6]. Also, a small number of studies deals with the optimization of battery consumption in electric bicycles. In this work, we will focus on the latter field of study, where the data used will be provided by different sensors and sources of external information, such as the altitude of GPS locations. This information will optimize the assistance provided by the engine in managing battery consumption; this will be done in accordance with route data and the user’s physical effort.

Despite the severe economic problems that Europe has experienced in recent years, the sale of bicycles has increased. In Spain, this increase was significantly high, according to the annual sales report of the Spanish Brands and Bicycle Association (AMBE—Asosciacion nacional de Marcas y Bicicletas de España) [7], with an average annual increase of over 10%, reaching 946.36 million euros in 2015, 11.43% more than in 2014. In particular, the report highlights an increase in the sale of electric bikes. As shown in Table 1, the percentage increase of electric bikes sold was +39.35% in 2015 (in 2014, 17,656 units were sold).

Despite the low number of electric bicycles sold (24,604 as opposed to 513,714 mountain bikes in 2015), a future where e-bikes become a real form of alternative transport is approaching. The use of a conventional bike is definitely the most energy-optimal and economic solution, but only works for traveling short distances (whether it is a mountain, road or city bike) and is only convenient on flat surfaces. Unlike traditional motor vehicles, the energy consumption of an electric bicycle is considerably lower. If we compare a traditional bicycle with an electric one, an electric bicycle certainly provides the user with greater comfort when travelling, regardless of the distance and the slope of the road Many countries have introduced laws regarding the use of e-bikes, and in countries such as Spain, Finland and the United Kingdom, the top speed cannot exceed 25 km/h and the motor’s maximum power output cannot exceed 250 Wh [8]. This type of bicycle has a pedal assist system, also called PAS or Pedelec [9]. The PAS incorporates a sensor which primary function is the detection of the speed and force transmitted to the pedals. When the user applies pressure to the pedal a unit of embedded control in the frame of the bicycle records this signal, providing the required power to the engine. This energy is used by the engine in order to reduce the user’s physical strain. When the user stops pedaling or presses on the brakes, the engine enters into a state of inactivity. The configuration of the different levels of assistance in an electric bicycle depends on the manufacturer. The assistance levels can be controlled by the user via two buttons on the handlebar, one to increase the assistance level of the engine and the other to decrease it. These levels can be managed automatically by the inclusion of sensor and actuation systems. Automatic management will intelligently manage the engine, thus it will manage power consumption.

In recent years, significant advances have been made in sensing systems and wireless sensor networks [10], which have resulted in phenomena such as the internet of things (IoT) [11], that allows us to connect everyday objects permanently [12]. Electric bicycles also incorporate sensors and actuators that allow them to obtain information and modify their behavior. The collected information can be fused with information from other sources such as a heart rate monitor, GPS or altitude maps. Information fusion, and in particular multisensor data fusion, is key in the design of these new systems. They represent a field of transversal research in very important areas such as defense, robotics, automation, intelligent systems design and pattern recognition [13]. Information fusion is therefore fundamental in the design of systems based on WSN (wireless sensor networks), as described in this work.

The proposed architecture is designed from the standpoint of vehicles connected to a smart city. Thus, it is an innovative architecture for electric vehicles, in particular electric bikes, in mobile sensing environments. The concept of sensing is further developed in this work, by incorporating mobile sensing in e-bikes. Hardware components and intelligent systems are integrated in the architecture for information fusion, route optimization and finally, energy saving. Moreover, a route optimization system is introduced; it consists of segmenting travel routes so that they are divided into a subset of connected segments. For each of the segments a level of assistance is established in order to achieve the overall goal of minimizing time and consumption on the route. An objective function is proposed to minimize the two conflicting factors: energy consumption and the time taken to complete each segment. The proposed architecture uses swarm intelligence techniques for the optimization of the objective function. One of the innovations of this proposal is that, being a nonlinear problem, it is not possible to use a traditional approach; therefore, this paper proposes the use of evolutionary algorithms in combination with neural networks to address this problem. Artificial neural networks are used to estimate the different parameters required by the evolutionary algorithm.

Energy saving is one of the key tasks in the development of sustainable and environmentally friendly cities. Thanks to the system developed in this work, a user who uses his electric bicycle to move from place to place on a daily basis, will find it very easy to save energy. A substantial optimization is achieved by increasing the range of the battery, without any significant increases in the time required to travel a route and in the user’s physical effort. Unlike conventional route optimization systems, the designed system does not provide an alternative route to the user. Instead, the system optimizes the selected route without changing its course. The user may not wish to travel the shortest or the most optimal way to reach his destination. There may be a variety of reasons for which a user may wish to travel a route of his choice, for example it may be safer due to the existence of a bike lane or they may wish to travel a route they already know.

This article is structured as follows: Section 2 reviews the current state of the art work on route optimization in electric vehicles; Section 3 describes the proposed system in detail; Section 4 presents the case study which was conducted to validate the proposed architecture and the results obtained. Finally, conclusions drawn from the work are outlined in Section 5.

## 2. Background

Route optimization for electric bicycles is a new field of research and at present no other studies have dealt with this issue in-depth, the studies found in the literature deal with hybrid electric vehicles (HEV) and 100% electric vehicles. They address the problems of estimation and optimization of fuel or energy. In [14], the authors propose a method for two-dimensional dynamic programming (DP). DP is combined with a novel route segmentation approach which minimizes fuel consumption on a pre-programmed route. The route is split into several segments that vary in size depending on the characteristics of the road (e.g., the speed on that road segment, traffic conditions and slope), this allows for higher efficiency. The same authors propose in [6] the use of a receding horizon control (RHC) algorithm [15] to calculate the set-point in order to optimize the state of charge (SOC) of the vehicle battery for a given route. In this paper, the original route, which has a large number of segments, is replaced by a virtual path with fewer segments, allowing the optimization calculations to be performed more quickly.

Recent research has focused on the physical models (model-based approaches) of vehicles and the environment. This is demonstrated in [16], where the authors develop a generic model with a variety of parameters for different types of vehicles. To determine an optimal route for the user, terrain topology data is obtained from Google Earth. Reference [17] takes a different, model-based approach in which Markov chains and particle filters are used to handle all non-deterministic situations such as traffic conditions or the user’s driving style. There are also many examples, such as [18], which focus on optimizing energy consumption for a particular route by examining the behavior of air conditioning systems and how they affect consumption.

Other studies focus on analyzing users’ driving data in order to identify and model the factors affecting the observable dataset. These datasets are obtained from different types of sensors deployed in most electric vehicles, as well as the data provided by smartphones. An example of this can be seen in [19], where the authors propose a linear regression model that uses speed, elevation and real-time consumption data (online), to provide an accurate estimate of energy consumption. In [20], the authors propose a social system to perform a crowdsourcing prediction of fuel data consumption for routes that have not been previously travelled by the user. In this paper, the authors use consumption and travel data from thousands of users and compare them with the user consumption profile to identify similar drivers. Using a linear regression model, the user’s consumption can be estimated based on the consumption data of users with a similar profile. As for studies that are completely focused on electric bicycles, in [21] the authors evaluate two prediction methods in order to obtain the driving range of an e-bike. They do this by first applying a prediction model mean, and then by evaluating a linear regression model. The authors obtained data from different users through a series of sensors deployed in an e-bike in combination with other data sources such as the users’ mobile devices or external data sources such as OpenStreetMap. Specifically, from the sensors deployed in the e-bike; they obtained voltage, current and battery temperature data. From the users’ mobile devices, they obtained the GPS signal, acceleration and gyroscope data. OpenStreetMap provided data on traffic signs and traffic lights located on the route. In this work, prediction models are used in cases where the final destination of the route is unknown, and only historical data on consumption are known. On the other hand, the use of linear regression models is considered in cases where the users’ final destinations are known. Specific models are applied for the type of road and for features such as traffic and the number of traffic lights.

There are several studies that propose approaches to address information fusion in multi-sensor systems in the current literature [22,23,24,25,26,27]. There are also works that address multi-sensor information fusion in the automotive industry [28], where the authors propose a multi-sensor architecture for the fusion of delayed observations applied to vehicle localization. The authors of [29] propose an architecture based on information fusion for the detection of pedestrians from motor vehicles. In [30], the authors propose an information fusion system for the implementation of a vehicle localization system in urban areas. There have been no works of this kind for electric vehicles, especially electric bicycles.

After examining the current state of the art research, we have discovered a research gap regarding studies of electric bikes that would deal with resource optimization on routes. Most of the existing projects focus on electric cars, which have very different characteristics to electric bicycles and their optimization goals are different. For example, in an electric bike, the optimization of the level of assistance provided by the electrical engine to the user is essential along the different segments of a route. Electric bicycles have recently been introduced and we have noticed that there are no relevant studies aimed at optimizing electricity consumption. Although evolutionary algorithms and swarm intelligence have been used in many optimization studies, it has not been possible to find studies focusing on optimization problems in electric bikes with assist levels. The continuous development of the e-bike market makes it necessary to provide innovative solutions to the users of these vehicles. Therefore, in this article we propose an architecture focused on route optimization in electric bicycles with an approach based on swarm intelligence algorithms.

## 3. Proposed Architecture

The aim of this paper is to design a mobile sensor architecture able to optimize a regularly travelled route by a user-assisted electric bicycle. This architecture is composed of a sensor network embedded in the bicycle, as well as wearable sensors and sensors deployed in the user’s smartphone. The hardware includes a development board for the management of the engine and the monitoring of the battery. When a route is being travelled all the data generated by these sensors are sent by the smartphone to a remote cloud server, where they are fused with geographical data in order to optimize energy consumption without increasing user effort and without reducing comfort.

**Figure 1:**
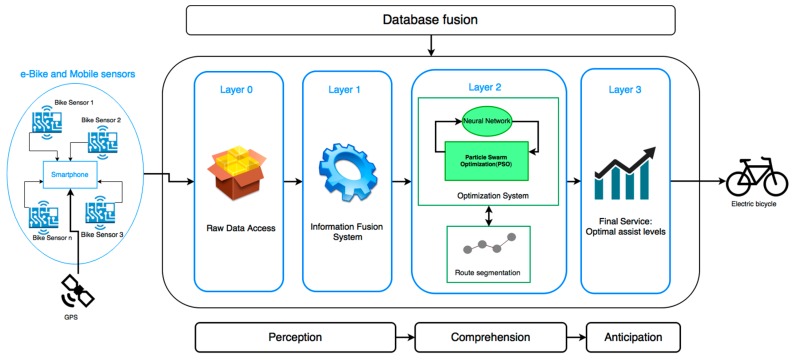
Data fusion architecture diagram proposed.

This paper proposes an architecture for the fusion of geographic data and the data collected by the deployed sensors. This architecture is based on the revised version of the original JDL (Joint Directors of Laboratories) model [31]. Figure 1 shows the information fusion architecture designed in this work, divided into four layers:

- ***Layer 0***: The architecture facilitates the integration of the different sensors, regardless of their communication technology. This is because the layer implements encapsulation mechanisms for the raw data received from different communication protocols both wireless (WiFi, Bluetooth or ANT+) and wired (controller area network (CAN) bus). In this way, the architecture is able to obtain data from new sensors connected to the system. Layer 0 corresponds to layer 0 of the revised JDL model and allows raw data from e-bike journeys to be obtained. As for the upper layers of the architecture, they are able to obtain information from any of the sensor networks.
- ***Layer 1***: The raw data collected by the different sensors in layer 0 are pre-processed in this layer. Once the data from heterogeneous sources are obtained, information fusion algorithms are performed and a new entity is defined to represent the knowledge. To model this entity, the RDF (resource description framework) language is used, which allows for standard definitions of entities and knowledge reuse capacities.
- ***Layer 2***: In this layer, levels 2 to 4 from the JDL model for information fusion are integrated. The optimization mechanisms are implemented on the basis of the information merged in the previous layer. The optimization model incorporates information from segmented routes, geographic characteristics of the routes, previous consumption data, information from bicycle sensors, wearable devices and mobile sensors. As an output of the optimization models, assistance levels are proposed to the user according to the characteristics of the route travelled, the battery level and the physical strain of the user. The optimization is based on particle swarm (PSO), which is backed by a neural network in order to optimize several parameters in the routes.
- ***Layer 3***: It is the top layer of the architecture and its purpose is to provide a series of end services through the merging of the information produced in the lower layers. This layer includes the latest levels of the revised JDL model that provide services through a human–machine interface (HMI). In the case of the proposed architecture, the result obtained after the analysis and data processing in layer 2, is a vector of optimal assistance. This vector is sent back to the user’s smartphone, which will be in charge of operating them automatically in the course of a new route optimized by the same path.

**Figure 2:**
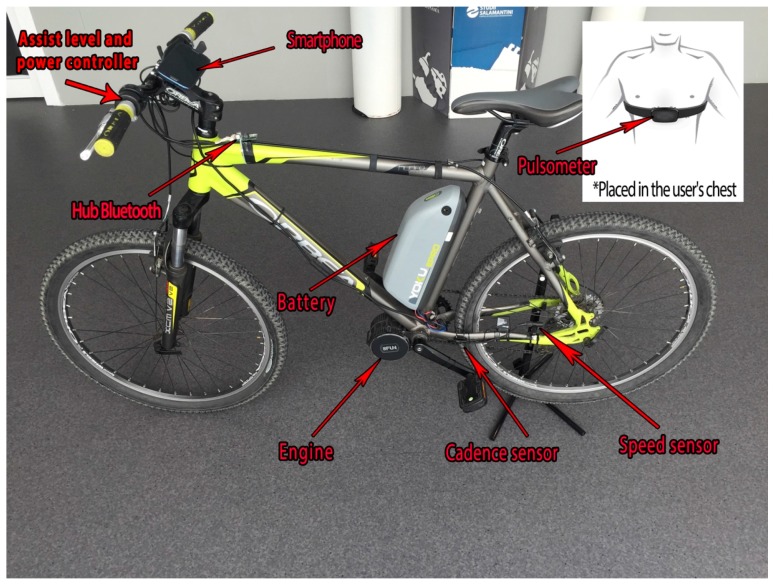
Sensors and e-bike used to collect data.

The sensor network deployed in the architecture is the most commonly used for electric bikes and practically all of the commercial e-bikes already have them. When a user travels a route on an electric bike, it is important that certain parameters are registered during the ride to provide useful information for route optimization. Figure 2 shows a diagram of the sensors used in the design of the architecture.

The wheel’s speed sensor is based on the functioning of the Hall effect sensor, which detects the rotation frequency of a magnet that is placed on one of the spokes. The cadence sensor measures the frequency the user is pedaling with, and it is also used as a supplementary sensor for measuring speed. For real-time heart rate monitoring, the user carries a heart rate monitor while on the e-bike. This device consists of an elastic strap with an electrode attached which is placed on the central part of the user’s chest. This device is also connected wirelessly via a Bluetooth connection. The engine (with a battery) is the main part of an electric bike. In the case of the engine that was used in this case study, it provides a number of parameters that are registered by the different internal sensors. These parameters are detailed below:

- **Assist level**: The engine provides information on the level of assistance that the user has selected through a control located on the handlebar. This control has the ability to turn the system on and off.
- **Remaining battery level**: Also called state of charge (SoC). The battery is connected to the engine, so that its sensors can read the different values. The remaining battery level provides a percentage estimate of the energy remaining in the battery.
- **Instant battery voltage**: The engine gives the battery voltage at every instant.
- **Instant current of the battery**: The voltage, also issues the current.
- **Temperature**: Battery and engine temperature can be checked at any moment.

**Figure 3:**
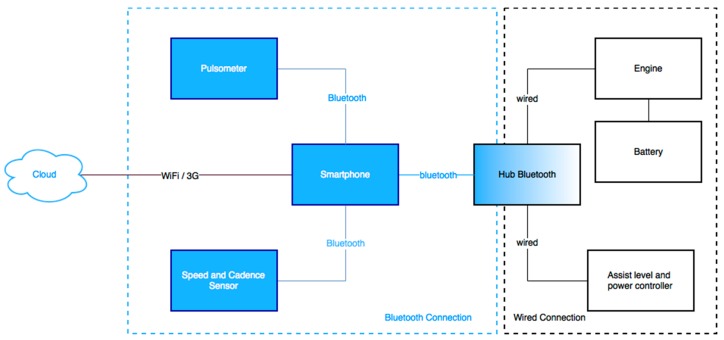
Device connection scheme.

**Figure 4:**
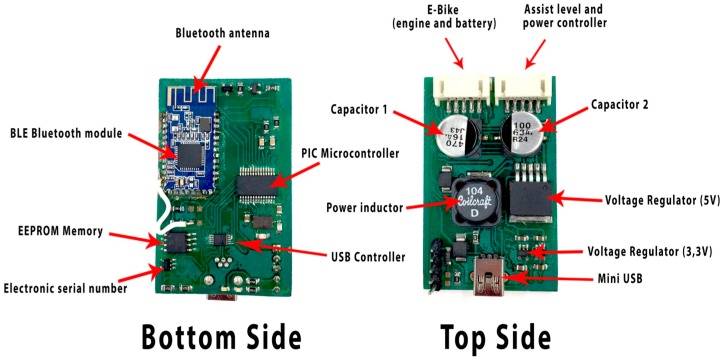
Hub Bluetooth components.

The information described above is provided by the engine, and together with the information from the battery, it is sent via data bus wiring to the Bluetooth hub. In Figure 3, the connection diagram of the different elements deployed in the sensor network can be seen. The purpose of the Bluetooth hub device is to send and receive data between the user’s smartphone and the electric bicycle. Figure 4 shows the device designed in this work, which can be integrated into a conventional e-bike as shown. Bluetooth 4.0 BLE (Bluetooth low energy) HC05 module sends all data to the user’s smartphone. These data arrive at the hub via two wired connections. In the first wired connection, the data for the engine and the battery of the bicycle are received. In the second connection, the assist level and power controller data sent by the handlebar controller are received. Orders are sent from this device for changing the level of assistance and turning the equipment on and off. It also has a PIC (programmable intelligent computer) microcontroller module responsible for performing computing operations. An EEPROM memory stores data and a driver chip DS28CM00 assures safety by providing a unique identifier to each of the devices. It also has a Mini USB (Universal Serial Bus) port to perform firmware updates of the device.

The smart mobile device (smartphone) has two functions in the system. First, the sensors installed in it are used, such as the GPS sensor, compass and accelerometer. Second, it acts as a central element of the system by capturing and processing all network data and storing them in a database of the mobile application. Once a route is completed by the user, the application that stores all the data inserts it into the remote database. This database will be used as source data in the case study of this work.

### 3.1. Route Segmentation

**Figure 5:**
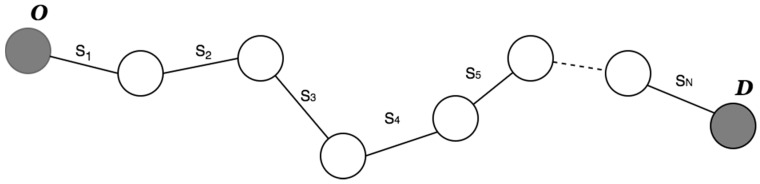
Route segmentation process.

One of the first challenges encountered is the division of a route into independent segments connected to each other. The purpose of this division is to reduce the complexity of the problem by assigning an optimum level of assistance to each segment. As described in the introduction of this paper, electric bicycles are operated through a series of assist levels which correspond to the amount of power supplied by the engine. At a higher level of assistance, increased engine power will be obtained, causing higher energy consumption. The criterion for this division is based on the slope difference at the different points of the route. For each of the GPS points p, knowing their slope data, it is possible to group the adjacent route points that have a slope magnitude $g_{i}$ similarity, in the same segment $S_{i}$. These calculated segments may have different $l_{i}$ lengths. Then, a route is created with origin O and destination D which is divided into a number of i=1,…,N segments $S_{i}$ connected with each other as shown in Figure 5.

**Figure 6:**
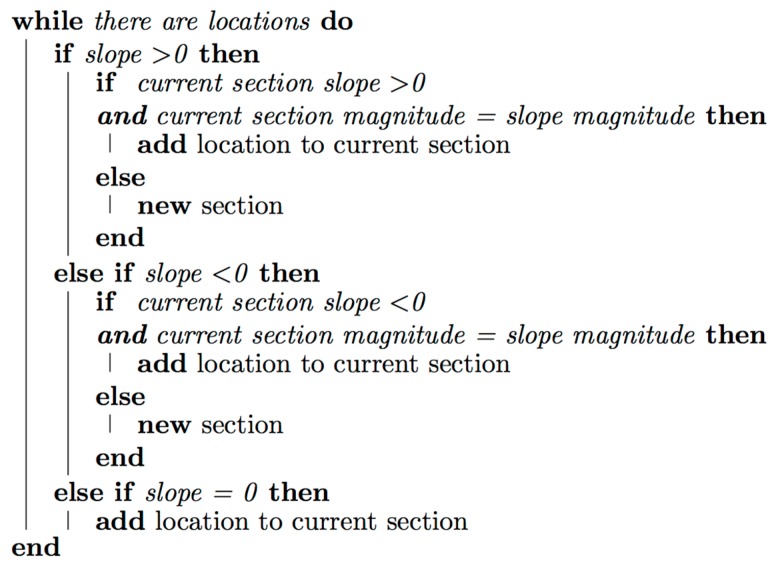
Route segmentation algorithm developed.

By grouping the points according to their slopes, we manage to adjust all of the calculated segments $S_{i}$ to the original profile. This grouping facilitates the allocation of levels of assistance based on the following premise: 

The higher the slope of the segment, the greater the user’s effort is to surmount it, and therefore, the higher the level of assistance required from the engine. The first approach of this algorithm groups the segments according to the sign of their points p (positive or negative), considering only the sign of the slope of the previous point. However, the main disadvantage of this approach is that is does not provide a realistic representation of the route, since within a segment of a positive slope, for example, there may be segments that are steeper than others, thus it should not be only considered whether the slope is positive or negative. Magnitude must also be considered, hence, the slopes that are 0% are grouped as positive or negative depending on the adjacent slopes. The pseudocode of the second approach is shown in Figure 6.

**Figure 7:**
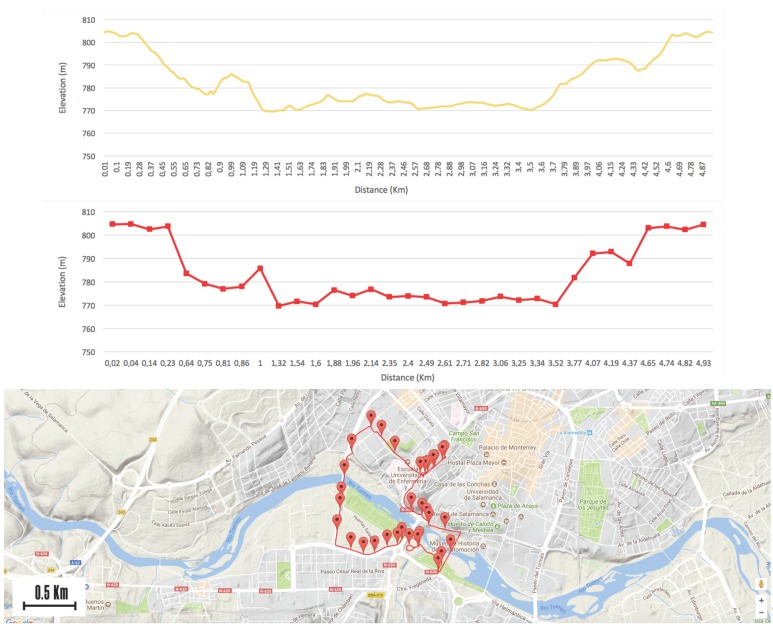
Example of route segmentation.

In Figure 7, the output of the algorithm for a 5 km route is shown. The first graph, with the yellow line, belongs to the route profile based on its altitude. In the second graph, we can see the different segments calculated by the algorithm. The red squares represent the starting points and the ending points of the different segments. At first glance, we can see that the segments which have been calculated correspond to the slope profile of the route. Finally, in the lower part we can see the different starting and ending points of the calculated segments shown on a Google map, the original path is represented with a red line.

### 3.2. Energy Consumption Calculation

At this point, the process of estimating battery consumption on a route is specified on the basis of the data captured by multiple sensors, which are described in the Proposed Architecture section. Considering that the voltage and ampere-hours for the user’s battery are known, we are able to calculate the total “fuel” available when the battery is charged to 100%. Voltage and current are data that are supplied by the engine at every moment during the completion of a route and their values decline over time. At once, the remaining battery levels decrease.

At the beginning of the journey, the user does not always have the battery charged to 100%. The charge value is calculated at the starting point of the route, so that it can be used as a reference for measuring consumption. As the battery level decreases, the voltage also decreases and the value of charge declines over time. However, in reality the values that are collected are not constant and may contain mistakes due to the fast voltage drop caused by a prompt application of the motor’s high power, at the request of the user. In order to obtain the range of a given electric bike, it is necessary to apply the range Equation (1) for a given e-bike, in this case we show the calculation of range for the e-bike used in this study.

(1) Range (Km)=V·q·Max. speed/Engine power

where V is the voltage in volts, q is the charge of the battery in Ah, Max. speed is the maximum speed in km/h and Engine power is the power of the engine in watts. The basic characteristics of the battery and the engine are described in Table 2:

Using a range Equation (1), the bicycle’s average speed is estimated to be 25 km/h (maximum speed established by the law) for this engine configuration + battery (in ideal conditions) in Equation (2):

Range (Km) = (36 V∙13 Ah∙25 km/h)/250 Wh = 46.8 km
(2)

Given that the values of the battery are nominal, and in reality it is dangerous to reach the consumption limits of the battery as explained in various studies [32], a correction factor is estimated from the total kilometers to a 70% range value, which is obtained in order to prevent the system from becoming damaged, as shown in Equation (3).

Range (with a security margin) = 32.76 km
(3)

Afterwards, we calculate the distance needed to consume 1 Ah at a maximum speed of 25 km/h (Equation (4)):

Kilometers for consuming 1 Ah = Range/Q (battery) = 32.76 km/13 Ah = 2.52 km/Ah
(4)

The resulting time is expressed in Equation (5):

Time taken in consuming 1 Ah (maximum power) = 2.52 km/25 km/h = 0.1008 h = 362.88 s
(5)

In this work, we try to minimize two different factors: consumption and the time taken to travel the routes. It should be taken into account that these two aspects are conflicting, therefore we must define an objective function that takes both aspects into account. This function intends to relate the amps consumed with the time spent on consuming one ampere in order to be able to assess the same units. The objective function for minimizing has to take into account both aspects and is defined in the following way (Equation (6)):

(6) $$f\left(t,q\right)=\frac{\sum t_{s}}{k}+\sum q_{s}$$

where, $\sum t_{s}$ is the sum of the time values in seconds of the different route segments and $\sum q_{s}$ is the sum in ampere hours of the different consumptions for each one of the segments. *K* is the time needed for consuming 1 Ah at maximum power, which is the previously calculated constant. The following rule should be applied to this Equation (7):

(7) $$\sum q_{s}<\mathrm{Battery}\;\mathrm{charge}$$

Once the objective function is defined, it is necessary to find a method to resolve it. Both time and consumption oscillate depending on assistance levels, therefore time and consumption must be estimated for each level of assist. This makes the problem unsolvable by linear programming as the functions that relate the two concepts are not linear. Since the relationship between input and output variables is not linear, neural networks will be used to estimate time and consumption.

### 3.3. Optimization Algorithm

When optimizing the function for finding the minimum values for time and consumption, as described in the previous section, we propose the use of swarm intelligence techniques. These techniques are specially designed to achieve optimal values for complex problems. Among all swarm intelligence algorithms, several were considered when conducting the proposed system, such as the ant colony optimization algorithm, which main objective is to find optimal paths in a given graph. This problem is comparable to the search for alternative paths that optimize fuel saving for a vehicle, however, it cannot be compared to the problem posed in this paper. We also considered the bee colony optimization algorithm, which proposes calculating optimal solutions through the behavior of individuals within a social group. Finally, the proposed algorithm for the minimization of the objective function was the particle swarm optimization algorithm. In this algorithm, in the proposed space of solutions, there is a population (swarming) of particles (insects) with S size. This population acts as search elements of the optimal solution and moves in the set of all the solutions, guided by the members of the swarm who have obtained the best positions (which are the best values for the objective function (*f*).

Each of these particles (*i*) communicates with its environment (g) and exchanges information on the best positions in the set of established solutions. The particles store information of the best position they obtain for themselves (local value) and the best position that is obtained for the group as a whole (global value). The information concerning the best position, which is stored, directly influences the behavior of the particle.

Therefore, each particle (i) stores the following values locally:

- $X_{i}$: is the current position of the particle
- $V_{i}$: speed associated with the particle (change of position)
- $p_{i}$: position of the best local solution found by the particle
- g: best position found by the environment of all the particles.

The basic version of the algorithm is the one proposed here and it is the most extended for the optimization of problems. As argued in [33], there have been improvements made in performance, parameter adjustment is easier, and behavior is more consistent when resolving different optimization problems in the most simplified versions of the algorithm. The main steps taken are described below:

- **Step 1:** Initially, a random position is assigned to the i particles, and $X_{i}\in {\mathbb{R}}^{n}$ is the range between the minimum and maximum values of the solution space proposed with $X_{i}=\left(x_{i1},x_{i2},\ldots ,x_{\mathrm{in}}\right)$, where n is the number of assist levels. It is also assigned a random speed within the limits set previously. Finally, the best local and best global random solution is stored.
- **Step 2:** After each of the iterations the speed must be recalculated on the basis of the speed and position of each particle. The formula that establishes the speed calculation at each new iteration is Equation (8):
  (8) $$V_{i}=\omega V_{i}+\phi_{1}\tau_{1}\left(p_{i}-X_{i}\right)+\phi_{2}\tau_{2}\left(g-X_{i}\right)$$
  where:
  - ω: is the parameter called “inertia” that controls the speed effect and prevents it from growing in an indefinite way
  - $\phi_{1}\;\mathrm{and}\;\phi_{2}$: are the values that mark the degree of confidence of the particle in itself and in the group
  - $\tau_{1}$ and $\tau_{2}$: random values between 1 and 0 have a stochastic influence on the velocity update.
  The new position of the particle once the new speed is calculated is Equation (9):
  (9) $$X_{i}=X_{i}+V_{i}$$
- **Step 3:** At this step updates of the best solutions will be made for each particle. In a matrix, the values of the best positions of each particle will be continuously stored, and in another matrix the best values of the objective function f. After each iteration, if the solution is calculated by the i particle, it is better than the best global solution known up until that moment, then, the value of g and the previous two matrices are updated. The g value is stored only when this situation occurs, and the current value obtained is better than the value stored in it.

Considering that the function being evaluated at each iteration of the algorithm is the optimization function f(t,q) (6), artificial neural network techniques are applied to calculate the value at each iteration. With all the data obtained from each of the times that the route that is to be optimized was travelled, two neural networks are trained to estimate the parameters of $t_{s}$ and $q_{s}$ through optimal assist levels calculated by the algorithm at each iteration.

Inputs for the neural network responsible for the estimate of consumption values $q_{s}$ are described in Table 3.

The neural network inputs, which estimate the final speed value of the segment and of which the time value $t_{s}$ is subtracted, are described in Table 4. Depending on the initial and final speed of the segment, the time spent on the optimization function is calculated.

To perform the training, a multilayer perceptron is used, and in the intermediate layer or hidden layer neurons, 2n+1 neurons are placed, following the theorem described by Kolmogorov. The activation functions of the chosen neural network are sigmoidal functions and their values were scaled in the range of [0.2–0.8]. To perform the estimation and training phase associated with the multilayer perceptron output layer, the following formulas were used. The formulas for the estimation of neurons in the intermediate layer and training are indicated in the work [34].

Equation (10) is used to perform the estimation for neuron *k* in the output layer. Where $y_{k}^{p}$ represents the output neuron *k* with respect to the pattern p, $f_{k}$ the activation function of neuron *k* of the output layer, *n* the number of entries in the neural network, $w_{kj}$ the weight binding the neuron *j* of the intermediate layer to the neuron *k* of the output layer, $y_{j}^{p}$ is the output of neuron j from the intermediate layer, and $\theta_{k}$ is the bia of neuron *k* for the output layer.

(10) $$y_{k}^{p}=f_{k}\left(\overset{2n+1}{\underset{j=1}{\sum }}w_{kj}\left(t\right)y_{j}^{p}\left(t\right)+\theta_{k}\right)$$

To estimate the updating of the weights and bias, the formulas indicated below are followed. The variables that are not defined above are η, the learning rate; $d_{k}^{p}$ the output for neuron *k* of the output layer; and μ momentum.

(11) $$w_{kj}^{p}\left(t+1\right)=w_{kj}^{p}\left(t\right)+\eta \left(d_{k}^{p}-y_{k}^{p}\right)\left(1-y_{k}^{p}\right)y_{k}^{p}y_{j}^{p}+\mu \left(w_{kj}^{p}\left(t\right)-w_{kj}^{p}\left(t-1\right)\right)$$

(12) $$\theta_{k}^{p}\left(t+1\right)=\theta_{k}^{p}\left(t\right)+\eta \left(d_{k}^{p}-y_{k}^{p}\right)\left(1-y_{k}^{p}\right)y_{k}^{p}+\mu \left(\theta_{k}^{p}\left(t\right)-\theta_{k}^{p}\left(t-1\right)\right)$$

## 4. Case Study and Results

This work was performed within the framework of the eBikeMotion [35] project, which focuses on the design of smart electric bikes. This project, co-developed by the University of Salamanca and the company StageMotion [36], has more than 5000 registered active users, most of them in the city of Salamanca (Spain). In order to validate the system proposed in this paper, a case study was carried out, which contains real data from routes travelled by e-bike users. These data were generated by 187 participants between the ages of 17 and 58, who were registered in the architecture and the app designed by the eBikeMotion project. Data was collected over a period of 11 months during the years 2015 and 2016. Initially, a route taken by 187 users at different times was selected, obtaining the average values that are presented in Table 5. This route is a well-known, 6 km route between the Santa Marta de Tormes town and Salamanca’s city center. Daily, this route is travelled by hundreds of citizens who live in Santa Marta de Tormes and work in the city of Salamanca. There is a bicycle lane for this route, making travel easier and safer for users. The 3.82 km segment that was considered in this study, was the most commonly travelled distance by the participants of this study.

**Figure 8:**
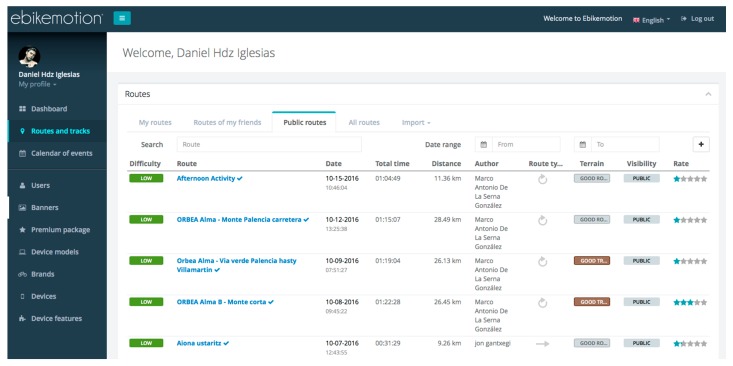
Screenshot of the eBikeMotion backend.

The mobile application registers the location data (GPS) together with all the data measured every 2.5 s by the sensors, thus, in the course of the study an average of 274 measurements were recorded for each user during the trip. The data storage and the subsequent analysis were performed on the remote server and a backend is provided to users, allowing them to view the results on the web. Figure 8 shows a screenshot of the web platform of the eBikeMotion project.

**Figure 9:**
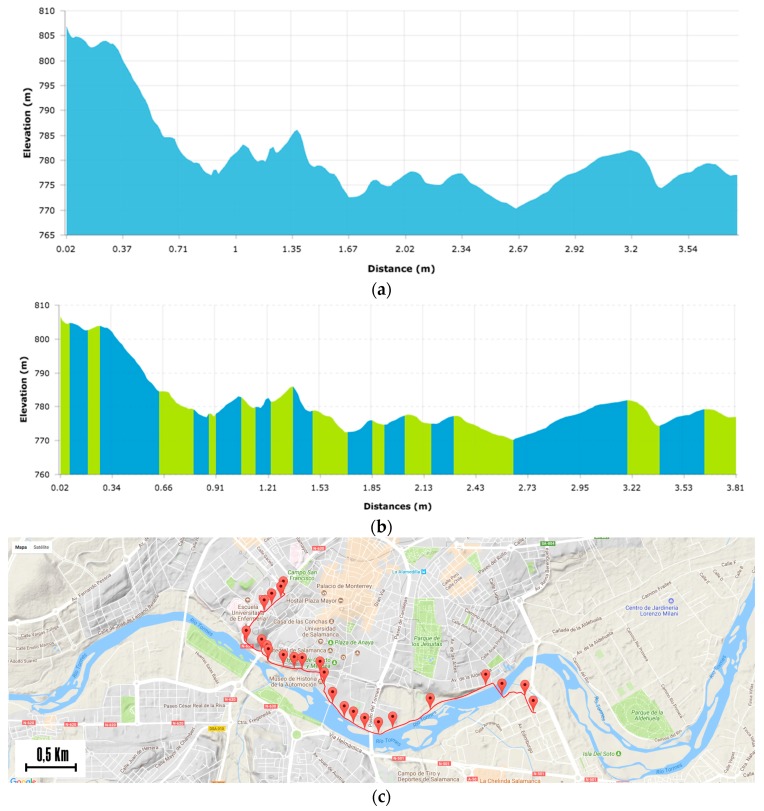
Route data for the case of study: (**a**) Route profile; (**b**) Division of the route into segments; (**c**) Location of the segments on Google Maps.

The first step of the process was to divide the route into different segments. Figure 9 shows the route profile Figure 9a and the division of the route into 23 segments (Figure 9b), which clearly match. Figure 9c shows how the calculated segments are corrected and adapted to the terrain.

Then, the training of the two neural networks was carried out, as it was necessary to estimate the values of consumption $q_{s}$ and time $t_{s}$. The data used for training the networks were obtained from each route location. The two neural networks had the same configuration as described in Section 3 and inputs and objectives as described in Table 3 and Table 4. Table 6 shows the data obtained after performing the training of the neural networks.

**Figure 10:**
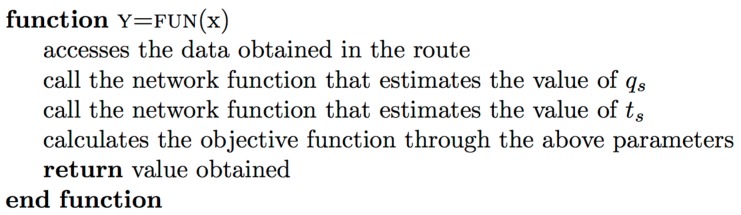
Objective function algorithm.

Then, the particle swarm function is applied, as described in Section 3.3 Optimization Algorithm. This function is used to perform the experiment of this case study. The function inputs are:

- *FUN*: The objective function described below in Figure 10.
- *nvars*: the number of segments necessary to calculate assist levels, in this case 23 segments
- *lb:* the lower limit of the search set, in this case, level 0 of assistance
- *ub:* the upper limit of the search set, in this case, level 9 of assistance.

The pseudocode of the function FUN is described in Figure 10.

The final function evaluated was Equation (13):

(13) x=particleswarm(FUN,nvars,lb,ub)

Once the particle swarm algorithm was executed, the output was a vector of the assist levels that corresponded to the values of the most optimal global positions of all the particles at the end of the execution of the function.

To validate the data obtained in this case study, the same route was repeated, and the obtained optimal assist levels were applied to the route in order to check if battery consumption was lower. For this reason, the starting points of each segment and the associated assist levels were downloaded to the mobile application on the user’s smartphone. After finishing the route, the data shown in Table 7 was collected.

**Figure 11:**
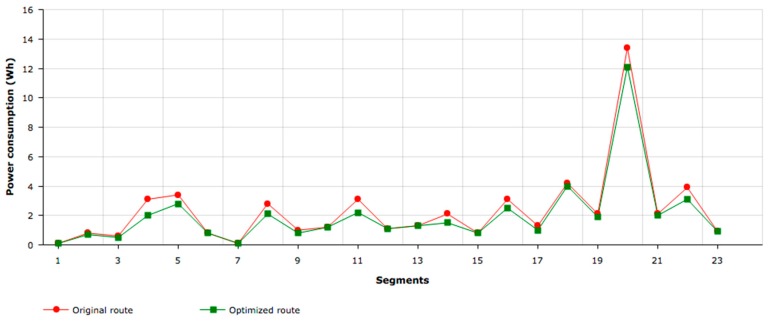
Consumption on the route before and after optimization.

As can be seen in Table 6 and Table 7, the distance of the experimental route is almost the same as the optimized experimental route (3.82 km and 3.88km, respectively), since the route is the same. However, in the optimized route, the duration is somewhat higher (12.6 min as opposed to 11.3 min). This occurred because the global assist level was lower, and kept the user’s heartbeat virtually constant; therefore, we can say that the relationship between assistance and user effort was correct. As shown in Figure 11, consumption is significantly reduced (10.32%), which in turn increases battery range. A greater reduction in the consumption of energy was achieved in segments where greater power is demanded. The segments with a higher power demand are those with a higher slope. Since user experience was affected by an excessive increase in physical effort at these segments (maximum heart rate was constant), the result was significant energy savings with the same experienced user effort and with a similar travel time.

On average, 10.32% of energy was saved, which means a total of 5.5 Wh per route. A user who travels the same route twice a day (to travel to and from work) saves 11 Wh in one day. This converts to around 220 Wh a month (doing the same route twice a day for 20 business days a month). As explained, an average of 47.8 Wh is required to travel this route. Therefore, with the energy savings made, the user will be able to travel for more than 4.5 extra routes per month with the same battery charge. Taking into account that it is a route of less than 4 km, 10.32% is a considerably high amount of energy saved. When operating this system on routes of greater distance or with greater slope than the route used in this case study, the savings will be even higher.

## 5. Conclusions

This work proposes an architecture that optimizes the use of electric bicycles. The architecture incorporates information fusion mechanisms that combine data from heterogeneous sources such as e-bike sensors, wearables, smartphone sensors and geographic information. In addition, it incorporates a new optimization mechanism, based on PSO and neural networks, which makes the e-bike assistance mechanism more effective by optimizing the different parameters.

Once the experimental phase of the study was finished, the application of the optimization methods resulted in a substantial decrease in consumption without any significant increase in the duration of travel. Hence, a greater battery range was achieved for the e-bike, enabling users to travel longer distances with the same battery charge. The main focus of this study was to optimize consumption, not other variables. However, if we wanted to give more importance to the time variable, this could be done by modifying the balance of the objective function. In future studies, we could look for ways to improve optimization results without increasing or even seeking to reduce the time taken to travel a route. Moreover, we will include some parameters in the objective functions in order to weigh time and consumption, so that the user can select different modes of behavior.

The use of neural networks allows the approximation of nonlinear functions that estimate consumption and speed, calculating time per segment. Artificial neural networks were used in combination with swarm intelligence algorithms to calculate the optimal result of the objective function. This technique was used because the objective function was not linear and therefore it was impossible to apply linear programming to solve the problem posed.

It is important to note that batteries and current measurement systems are not 100% precise at the time of calculating optimal consumption. Whilst working on this project, we encountered cases where two batteries with the same characteristics and the same manufacturer did not provide the same data under the same conditions. There are many variables that influence the behavior of these batteries, and their measurement and subsequent calculations should always be taken as approximate values and never as precise values. We would like to point to the importance of using artificial intelligence in the area of optimization of resources and energy. This field is becoming increasingly widespread and crucial, if we consider that our planet is running out of its energy resources.

As mentioned previously, in the current literature there are very few works that address this topic. It is necessary to continue working on the development of electric bicycle routes in future works, in order to achieve greater energetic sustainability in cities. Through the development of sensing systems and greater computational capacities of new smartphones, it is possible to improve future works concerning this type of optimization system. External servers will no longer have to be used, as we will be able to apply algorithms directly to smartphones. The use of mobile devices for information processing will make it possible to create a more easily scalable architecture, in which the economic cost of computing capacities will be reduced. As for the electric vehicle industry, it is also important that the physical capacities of batteries continue to improve, in order to be able to achieve better performance in combination with other systems, such as the one proposed in this work.

## Figures and Tables

**Table 1:** Data for bicycles purchased between 2014 and 2015 in Spain.

Year/Type of Bike	2014	2015	Evolution 14/15 (%)
Mountain	522,554	513,714	−1.69%
Road	56,638	68,273	20.54%
City	95,100	107,702	13.25%
Kid	396,600	389,546	−1.78%
Electric	17,656	24,604	39.35%
Total	1,088,548	1,103,839	1.40%

**Table 2:** Battery and engine characteristics.

Characteristic	Value
Electric charge (q)	13 Ah
Voltage (V)	36 V
Engine power (W)	250 W
Maximum speed (km/h)	25 km/h

**Table 3:** Network inputs for estimating consumption.

**Inputs**	Assist (calculated by the algorithm)
	Speed in the initial part of the segment
	Speed at the end of the segment
	User’s heartbeat (constant)
	Slope
**Objective**	Consumption (Ampere-hour)

**Table 4:** Network inputs for time estimation.

**Inputs**	Assist
	Speed in the initial part of the segment
	User’s heartbeat (constant)
	Slope
**Objective**	Speed at the end of the section

**Table 5:** Data of the route carried out in the case study.

Data	Value
Duration	11.3 min
Distance	3.82 km
Average speed	26.83 km/h
Maximum elevation	807 m
Minimum elevation	770 m
Average beat rate	93 bmp
Average rate	2.45 min/km
Gained elevation	43 m
Elevation loss	72 m
Total consumption	53.3 Wh
Maximum heart rate	112 bmp

**Table 6:** Neural network training results.

	Network to Estimate Consumption	Network to Estimate Speed
Training data	70%	70%
Data validation	15%	15%
Test data	15%	15%
Mean absolute error	9.7% of error	2.3 km/h of error

**Table 7:** Results obtained after data optimization.

Data	Value
Duration	12.6 min
Distance	3.88 km
Average speed	23.76 km/h
Maximum elevation	807 m
Minimum elevation	770 m
Average beat rate	98 bmp
Average rate	3.24 min/km
Gained elevation	44 m
Elevation loss	70 m
Total consumption	47.8 Wh
Maximum heart rate	116 bmp
